# Microbial community composition of *Pulsatilla chinensis (Bunge) Regel* root in different regions

**DOI:** 10.1515/biol-2025-1218

**Published:** 2025-12-31

**Authors:** Jun Shi, Gangjun Xi, Jiuling Ding, Hetong Yang, Chao Xu

**Affiliations:** Jiangsu Vocational College of Agricultural and Forestry, Zhengjiang, 202400, China

**Keywords:** *Pulsatilla chinensis (Bunge) Regel*, microbial community, root, 16S rRNA sequencing

## Abstract

This study investigated the microbial community composition of the roots of *Pulsatilla chinensis* (Bunge) Regel samples from three different regions. 16S rRNA sequencing of *P. chinensis* (Bunge) Regel samples from three different regions, Anhui (AH), Northeast China (DB), and Hebei (HB), was performed using Illumina high-throughput sequencing technology. Weighted gene co-expression network analysis (WGCNA) was employed to find collaborative bacterial modules, identify hub microbiota. Finally, the correlation between the hub microbiota was evaluated. The top 10 phyla in the three groups included Acidobacteriota and Actinobacteriota; while the top 10 genera were Alphaproteobacteria and Dongia. Gemmatimonadaceae and Gemmatimonadales, Rhizobiales and Alphaproteobacteria, Rokubacteriales and Methylomirabilia were significantly overrepresented in DB, AH, and HB samples, respectively. The brown and turquoise modules from WGCNA correlated strongly with regional groups (|*r*| > 0.5 and *P* < 0.05). Moreover, 14 hub microbiotas were identified at the species level. Among which, Gemmatimonadaceae was positively correlated with Proteobacteria, whereas Acidobacteriota was negatively associated with bacteriap25. The microbial community composition of *P. chinensis* (Bunge) Regel roots showed differences in the three regions, and the 14 hub microbiotas may be associated with the altitudinal, geographical, climatic, and cultivation practices of this species.

## 1 Introduction

*Pulsatilla chinensis (Bunge) Regel* is a buttercup plant with a long medicinal history in China [1], [2], [3]. Its main chemical components are paeoniflorin, lignans, and triterpenoid saponins, which are important in treating intestinal diseases [4], 5]. It has also shown good pharmacological properties, including anti-inflammatory, antibacterial, antitumor, and immune regulatory effects [6], [7], [8]. *P. chinensis (Bunge) Regel* and its active ingredients can inhibit the nuclear factor kappa-light-chain-enhancer of activated B cells (NF-κ B) signaling pathway, reduce the release of pro-inflammatory factors such as tumor necrosis factor (TNF)-α and interleukin (IL)-6, and induce the heme oxygenase-1/nuclear factor erythroid 2-related factor 2 antioxidant pathway to alleviate inflammatory damage [9]. Animal experiments have shown that *P. chinensis (Bunge) Regel* may have anti-tumor activity by inhibiting the growth and spread of tumor cells [9], 10]. In summary, *P. chinensis (Bunge) Regel* has high medicinal value, supporting its potential as a multifunctional therapeutic agent for immune inflammatory diseases and cancer-related syndromes.

Plant microbiota is a collective term for microorganisms living inside and outside a plant [11], 12]. It comprises various microorganisms, including bacteria, fungi, viruses, and protists [13]. Regional differences exist in plant root microbial communities [14]. The regional heterogeneity of soil physicochemical properties constitutes the core driving force for the differentiation of plant root microbial communities [15]. There are significant differences in soil pH, nitrogen, phosphorus, potassium content, water retention, and permeability in different regions, which directly regulate the metabolic adaptability of microorganisms. Climate factors such as temperature, humidity, precipitation, and altitude directly affect microbial activity and metabolic rate [16]. Terrain undulation indirectly leads to the regional differentiation of microbial communities by affecting soil water and heat distribution [17]. In addition, differences in the types of associated plants in different regions result in different root exudate components, which attract different microbial communities [18]. Moreover, human activities in different regions, such as agricultural measures and pollution stress, can drive the replacement of sensitive bacterial species with tolerant microbial communities by changing soil redox conditions, thereby causing changes in microbial communities [19], 20]. Plant microbiota is a vital part of the interaction with the environment and has a significant impact on plant growth and environmental adaptation by helping plants absorb nutrients, controlling diseases, and improving plant resistance to stress [21], 22]. Differences in plant microbiota are closely related to the pharmacological effects of their active ingredients [23], 24].

*Pulsatilla chinensis (Bunge) Regel* is widely distributed and mainly produced in Heilongjiang, Jilin, Liaoning, Inner Mongolia, Henan, Shandong, Shaanxi, Jiangsu, and other provinces. Given that *P. chinensis (Bunge) Regel* is widely distributed throughout China, its rhizosphere microbial community exhibits significant ecological differentiation owing to the differences in climate, soil, and vegetation types in different regions. A previous study discovered that the quantity of saponin B4 in *P. chinensis (Bunge) Regel* from 17 different production areas differed [25]. In addition, the relative abundances of *Actinobacteria/Firmicutes* was significantly positively correlated with saponin B4 content [26]. Signal molecules such as indole-3-acetic acid (IAA) and iron carriers secreted by rhizosphere microorganisms can activate the expression of secondary metabolism-related genes in *P. chinensis (Bunge) Regel*, thereby enhancing the efficiency of triterpenoid saponin synthesis [27], 28]. Nitrogen-fixing and phosphorus-solubilizing bacteria enhance the availability of soil nitrogen and phosphorus and promote the absorption of mineral elements by plants, thus enhancing the synthesis of medicinal components [29]. Differences in microbial communities in different regions may lead to variations in the content of active ingredients in *P. chinensis (Bunge) Regel*, further affecting its anti-inflammatory and anti-tumor efficacy. Therefore, it is of great significance to study the root microbiota of *P. chinensis (Bunge) Regel* from different regions.

16S rRNA sequencing can be used to identify and classify microorganisms and provide information on the composition and diversity of microbial communities [30], [31], [32]. In this study, the microbial community composition in *P. chinensis (Bunge) Regel* roots from three different regions was studied using 16S rRNA sequencing, and the associations between hub microbiota were further explored. This study offers new perspectives on the cultivation of *P. chinensis (Bunge) Regel.*

## 2 Materials and methods

### 2.1 Sample collection

We collected wild seedlings of *P. chinensis (Bunge) Regel* from three regions, Hebei (HB), Anhui (AH), and Northeast (DB), and planted them in the greenhouse (25 °C/18 °C day/night, 16 h light) of Jiangsu Agriculture and Forestry Vocational and Technical College. The plants were cultivated in local rural soil to ensure identical soil conditions. The research was conducted from March 2022 to March 2023. The plants were then removed from the flowerpot, large soil aggregates were manually removed, and the soil firmly attached to the roots was acquired using a sterile brush and treated as rhizosphere soil. First, the rhizosphere soil was sampled and sieved to remove plant debris. Subsequently, a portion of the soil sample was placed in a sterile centrifuge tube, flash-frozen in liquid nitrogen, and stored at −80 °C for subsequent analyses.

### 2.2 DNA extraction and 16S rRNA sequencing

DNA extraction and 16S rRNA gene sequencing were performed using established protocols optimized for root-associated microbial community analyses.

Root-adhering soil samples (500 mg fresh weight) were collected by gently shaking the roots in sterile PBS (pH 7.0) to dislodge the rhizosphere soil. Total genomic DNA was extracted using the DNeasy PowerSoil Kit (Qiagen, Valencia, CA, U.S.) following the manufacturer’s instructions, including bead-beating at 5.5 m/s for 30 s using a FastPrep-24 instrument to ensure efficient lysis of Gram-positive bacteria and spore-forming species. DNA integrity was confirmed by 1 % agarose gel electrophoresis, and concentration was quantified using the Qubit^®^ dsDNA HS Assay Kit (Life Technologies, CA, U.S.). DNA samples from the individual strains of each variety were used for 16S rRNA sequencing.

16S rRNA sequencing was performed using the IonS5™XL sequencing technology platform with the universal primers 515F (5′-GTGCCAGCMGCCGCGGTAA-3′) and 806R (5′-GGACTACHVGGGTWTCTAAT-3′), which amplified the V4 hypervariable region of the 16S rRNA gene. PCR reactions were performed in triplicate using Phusion High-Fidelity DNA Polymerase (Thermo Fisher Scientific, Waltham, MA, U.S.) to minimize amplification bias. Amplicons were purified with AMPure XP beads (Beckman Coulter, Brea, CA, U.S.) and sequenced on the IonS5™XL platform (Thermo Fisher Scientific) using 2 × 300 bp paired-end reads. To ensure high-fidelity microbial community profiling, we optimized the bioinformatics pipeline for the 16S rRNA gene sequencing data using rigorous quality control and classification steps. Raw reads were first trimmed to remove bases with Phred scores <20 and adapter sequences using Cutadapt (version 1.9.1) with a 0.1 error rate threshold, retaining only reads ≥150 bp in length. Chimeric sequences were then identified via UCHIME (version 4.2.40) in both *de novo* and reference-based modes, with sequences flagged in either mode discarded to minimize false negatives. Clean reads were clustered into 97%-similarity OTUs using UCLUST (version 1.2.22q) and assigned taxonomy against SILVA v138 via RDP Classifier (version 2.12) with an 80 % bootstrap confidence threshold, followed by contaminant filtering of OTUs detected in negative controls (PCR-grade water) at ≥ 0.1 % relative abundance using custom R scripts. The robustness of this pipeline was validated by sequencing the ZymoBIOMICS Microbial Community Standard, achieving 98.5 % species-level agreement with the expected composition, and rarefaction curves confirmed adequate sequencing depth for all samples, thereby meeting MIxS-BE standards for microbial community analysis.

### 2.3 Bioinformatics

This study comprehensively evaluated the microbial diversity, including alpha and beta diversity. The Chao1 index and Observed species were used to characterize richness, the Shannon and Simpson indices were used to characterize diversity, Pielou’s evenness index was used to characterize evenness, and Good’s coverage index was used to characterize coverage, in order to evaluate the alpha diversity of the three microbial communities. This study employed principal component analysis (PCA) and principal coordinate analysis (PCoA) to examine the beta diversity of the three microbial communities. Linear discriminant analysis effect size (LEfSe) was used to confirm the significant differences in the presence of microbes. Weighted gene co-expression network analysis (WGCNA) was used to identify highly collaborative bacterial modules. First, the top 15 % amplicon sequence variant (ASV) with large inter-sample variation was selected and analyzed using the R package “WGCNA” (Version 1.71) [33]. To satisfy the prerequisite of scale-free network distribution as much as possible, the power value at which the square value of the correlation coefficient first reached 0.85 was selected. The parameters were set using clustering and dynamic pruning methods to aggregate the bacteria with high correlations into modules. By calculating the correlation between modules and groups, modules that were closely related to the group (|*r*| > 0.5 and *P* < 0.05) were selected. In addition, the connectivity of the microbiota within key modules was calculated, and two criteria were utilized to screen the microbiota, including gene and key module significance >0.2, and module membership key module significance > 0.8. Cytoscape (version 3.6.1) was used to construct and visualize the interaction network of the microbiota between the key modules. The microbiota in the modules were compared and analyzed at the species level and the differences at the species level according to the annotation information to ASV, and the intersecting microbiota were considered as the hub microbiota. Spearman’s rank correlation coefficient was used to calculate the microbial abundance at the hub microbiota species level, and the correlation and *P* value between the hub microbiota were obtained.

## 3 Results

### 3.1 Change in bacterial diversity

**Figure 1:**
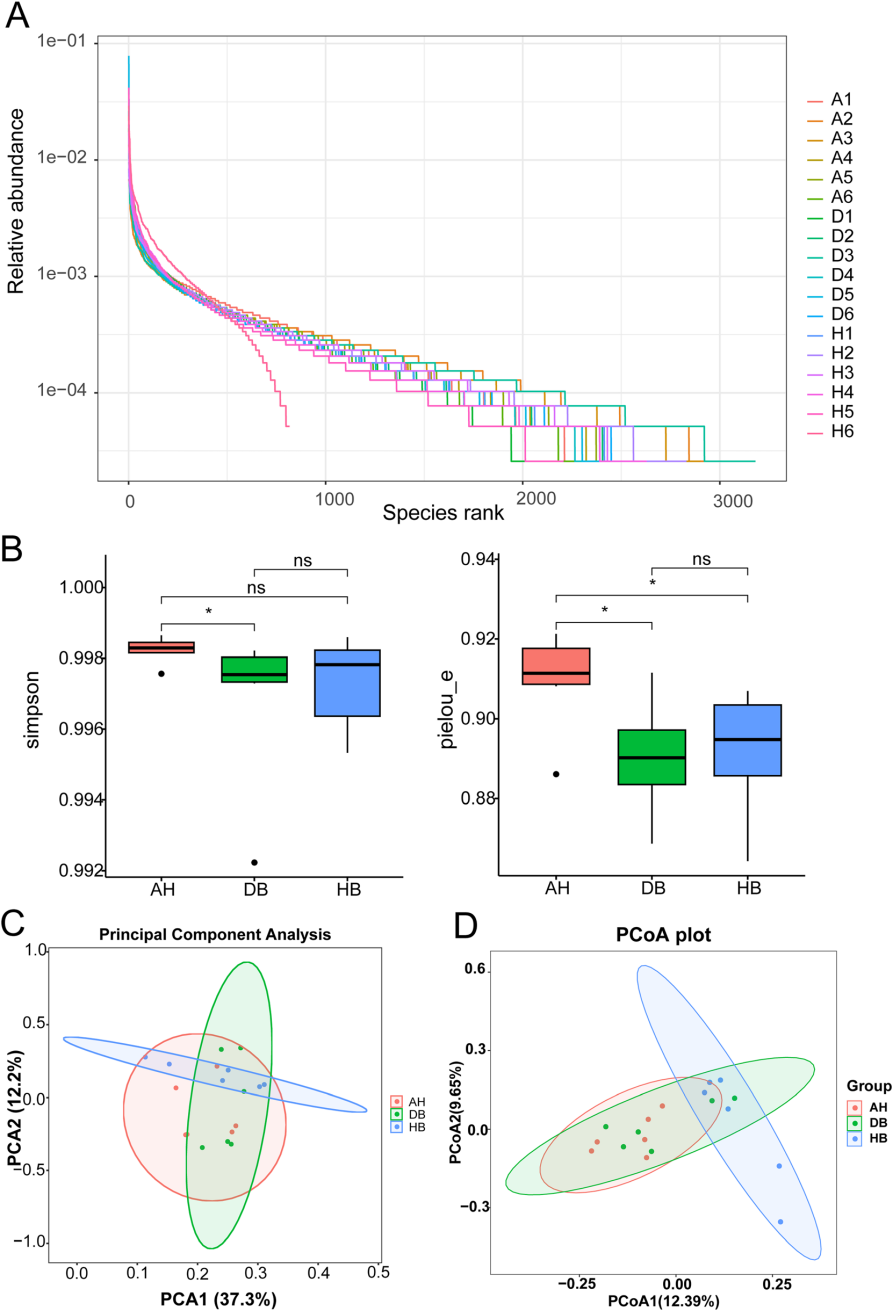
Change in bacterial diversity. (A) Rarefaction curves. Different colored curves represent different sample groups, with the horizontal and vertical axes representing species rank and relative abundance, respectively. (B) Alpha diversity analysis. The left image shows Simpson’s index, and the right image shows Pielou’s evenness index. (C) Principal components analysis (PCA). Each point represents a sample, and points of different colors correspond to different sample groups. The elliptical region represents the distribution range of each sample group, with PCA1 and PCA2 accounting for 37.3 % and 12.1 % of the variation, respectively. (D) Principal coordinate analysis (PCoA). The elliptical region represents the distribution range of each sample group, with PCoA1 and PCoA2 explaining 12.39 % and 9.65 % of the variation, respectively. Ns: *P* > 0.05, *: *P* < 0.05.

The rarefaction curve indicated the adequacy of all samples (Figure 1A). Alpha diversity analysis showed that the bacterial diversity in the DB group was greater than that in the AH group (*P* < 0.05; Figure 1B). PCA and PCoA showed different bacterial community compositions in the AH, DB, and HB groups (Figure 1C and D).

### 3.2 Comparison of the taxonomic profile of the microbiota

**Figure 2:**
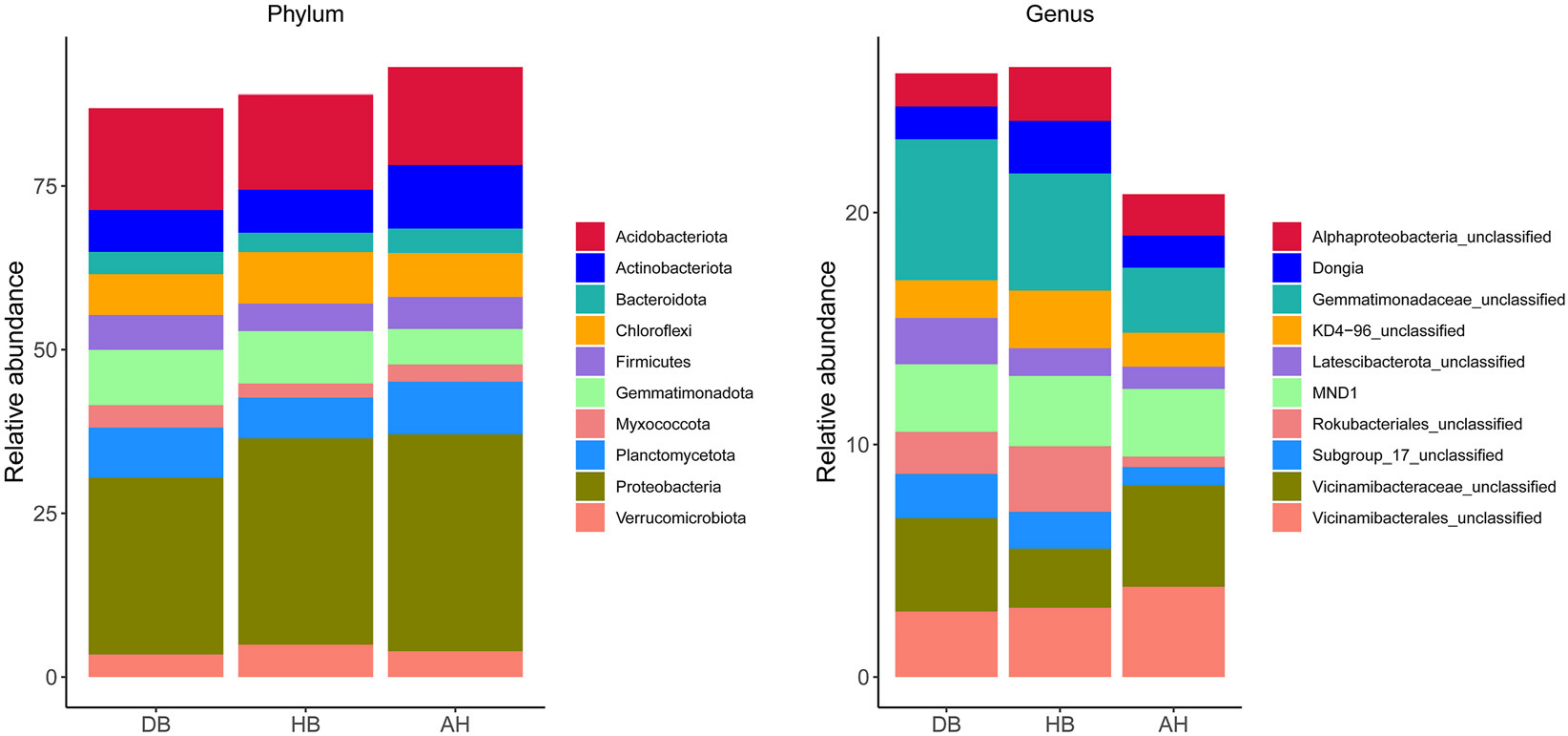
The relative abundance of bacteria at the phylum (left) and genus (right) levels. Each color represents a bacterial phylum (genus), and the vertical axis represents the relative abundance.

The relative abundances of the top 10 most abundant bacterial phyla and genera in the three groups are shown in Figure 2. The top 10 phyla in the three groups were *Acidobacteriota* (15.58 % in DB, 14.52 % in HB, and 14.92 % in AH), *Actinobacteriota* (6.44 % in DB, 6.61 % in HB, and 9.74 % in AH), *Bacteroidota* (3.40 % in DB, 2.93 % in HB, and 3.71 % in AH), *Chloroflexi* (6.17 % in DB, 7.85 % in HB, and 6.68 % in AH), *Firmicutes* (5.31 % in DB, 4.19 % in HB, and 4.88 % in AH), *Gemmatimonadota* (8.52 % in DB, 8.04 % in HB, and 5.47 % in AH), *Myxococcota* (3.43 % in DB, 2.15 % in HB, and 2.65 % in AH), *Planctomycetota* (7.55 % in DB, 6.25 % in HB, and 8.06 % in AH), *Proteobacteria* (27.05 % in DB, 31.53 % in HB, and 33.09 % in AH), and *Verrucomicrobiota* (3.46 % in DB, 4.89 % in HB, and 3.94 % in AH).

The top 10 genera in the three groups were *Alphaproteobacteria* (1.41 % in DB, 2.30 % in HB, and 1.79 % in AH), *Dongia* (1.42 % in DB, 2.27 % in HB, and 1.38 % in AH), *Gemmatimonadaceae* (6.07 % in DB, 5.04 % in HB, and 2.79 % in AH), *KD4−96* (1.64 % in DB, 2.49 % in HB, and 1.47 % in AH), *Latescibacterota* (1.99 % in DB, 1.19 % in HB, and 0.95 % in AH), *MND1* (2.92 % in DB, 3.05 % in HB, and 2.93 % in AH), *Rokubacteriales* (1.80 % in DB, 2.81 % in HB, and 0.43 % in AH), *Subgroup_17* (1.90 % in DB, 1.58 % in HB, and 0.78 % in AH), *Vicinamibacteraceae* (4.03 % in DB, 2.56 % in HB, and 4.38 % in AH), and *Vicinamibacterales* (2.81 % in DB, 2.97 % in HB, and 3.88 % in AH).

### 3.3 Dominant bacterial taxa

**Figure 3:**
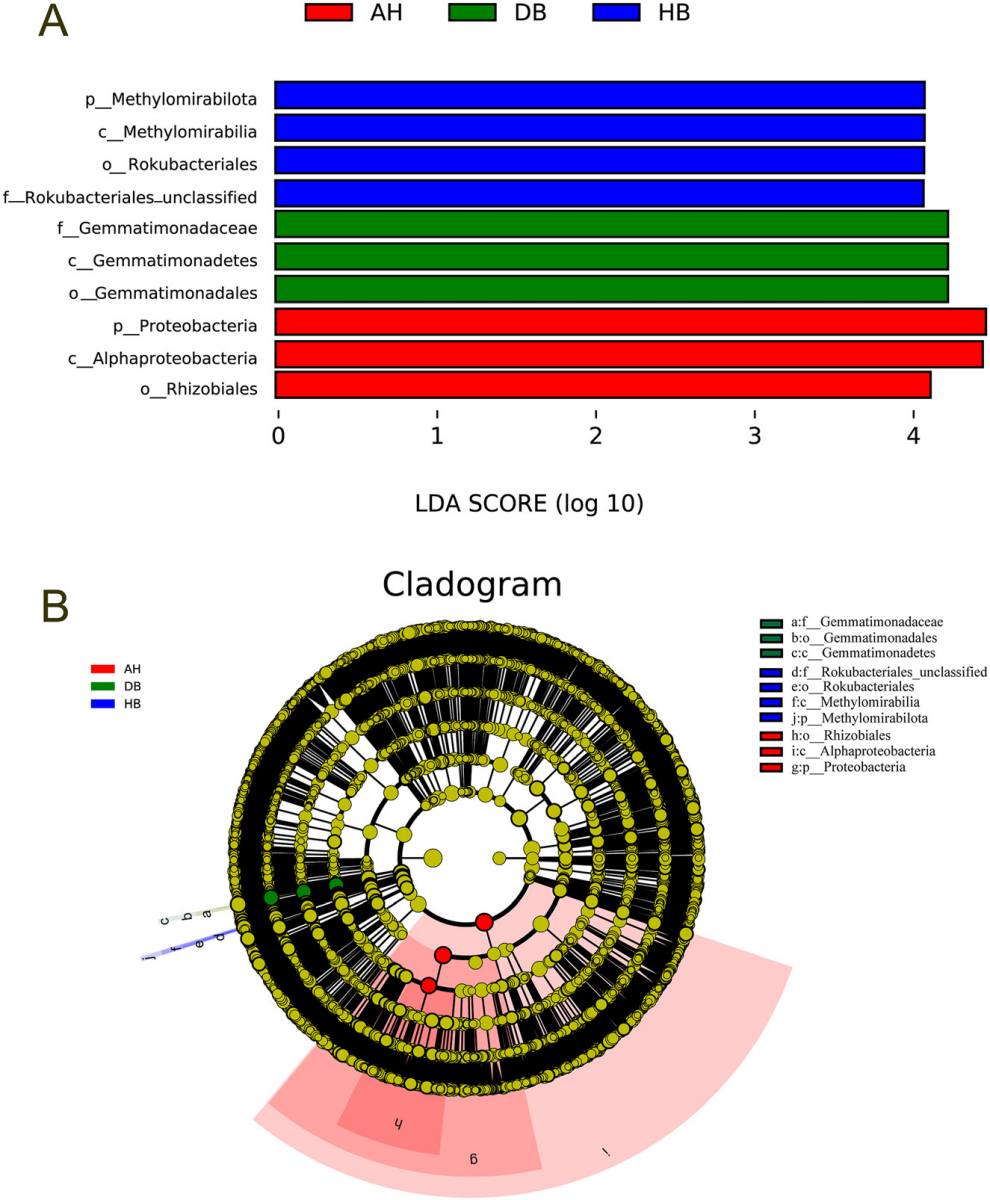
Differently abundant taxa identified using linear discriminant analysis effect size (LEfSe) analysis. (A) Cladogram showing differences in the relative abundance of taxa among the three groups. The plot was generated using the online LEfSe project. (B) Plot from LEfSe analysis. The length of the bar column represents the linear discriminant analysis (LDA) score. The figure shows microbial taxa with significant differences among the three groups.

LEfSe was used to generate a cladogram for identifying specific bacteria (Figure 3). Several bacteria, including *Gemmatimonadaceae*, *Gemmatimonadales*, and *Gemmatimonadetes* were significantly overrepresented in the DB samples; *Rhizobiales*, *Alphaproteobacteria*, and *Proteobacteria* were all significantly overrepresented in the AH samples; and *Rokubacteriales*, *Methylomirabilia*, and *Methylomirabilota* were all significantly overrepresented in the HB samples. These results further support our diversity results, showing large heterogeneity in the rhizosphere microbiota of *P. chinensis (Bunge) Regel* from different origins.

### 3.4 Identification of hub microbiota

**Figure 4:**
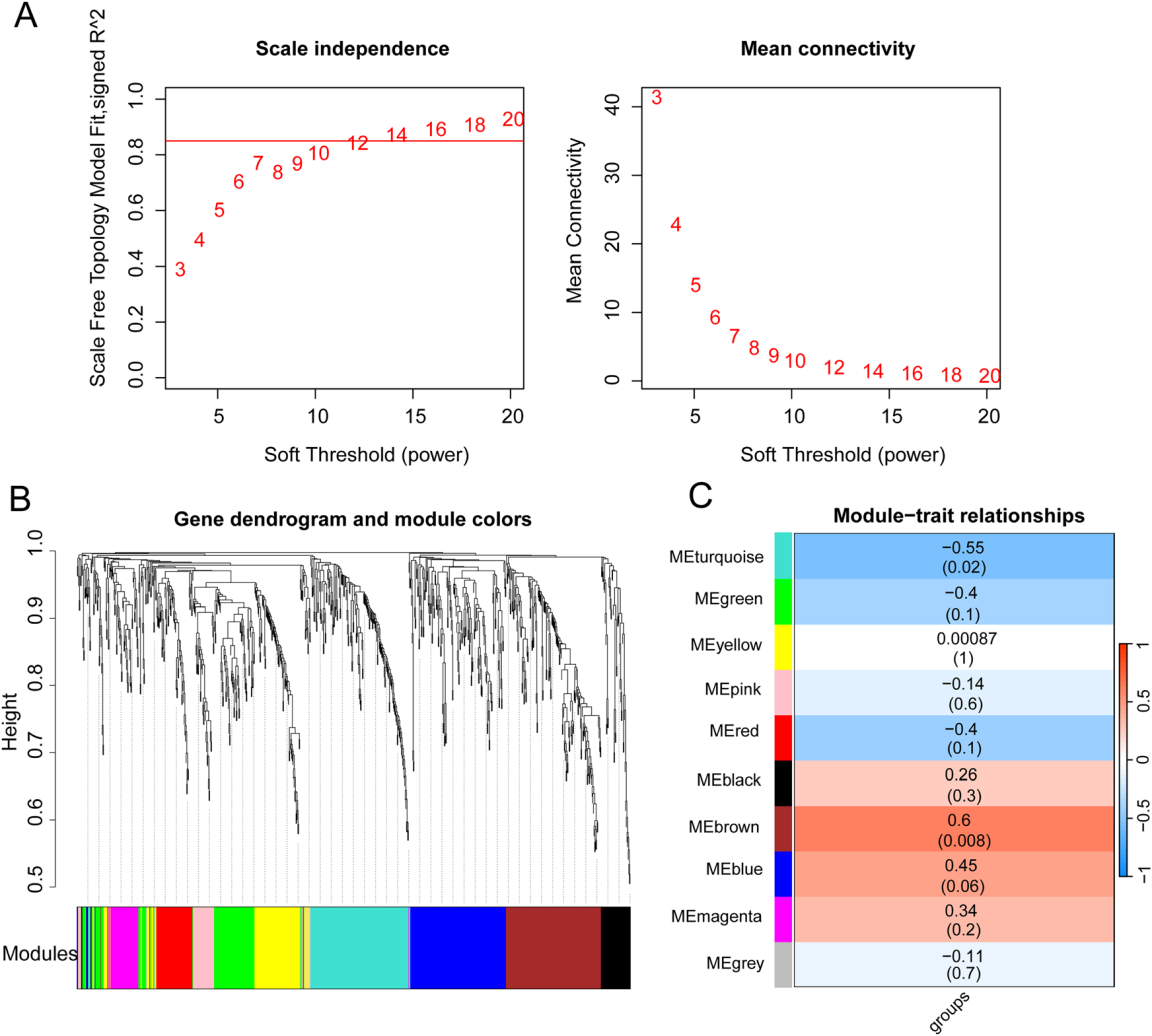
Weighted gene co-expression network analysis (WGCNA). (A) Hierarchical clustering of samples and selection of the weight parameter “power” of the adjacency matrix and the mean connectivity. The left figure shows the trend of the scale free topology model fit (R^2^) changing with an increase in the soft threshold, whereas the right figure shows the trend of average connectivity decreasing with an increase in the soft threshold. (B) The tree diagram of module division displays the hierarchical clustering relationships between genes, with colored bars at the bottom representing the different gene modules. Each module is composed of a set of genes with similar expression patterns, and color coding is used to distinguish the different modules. (C) Global outline of the relationship between the modules and regional groups. Colors represent the magnitude and direction of the correlation coefficients between different color modules, with red indicating a positive correlation and blue indicating a negative correlation. The numbers in each cell represent the correlation coefficient and corresponding *P*-value, helping to identify gene modules significantly associated with specific traits.

**Figure 5:**
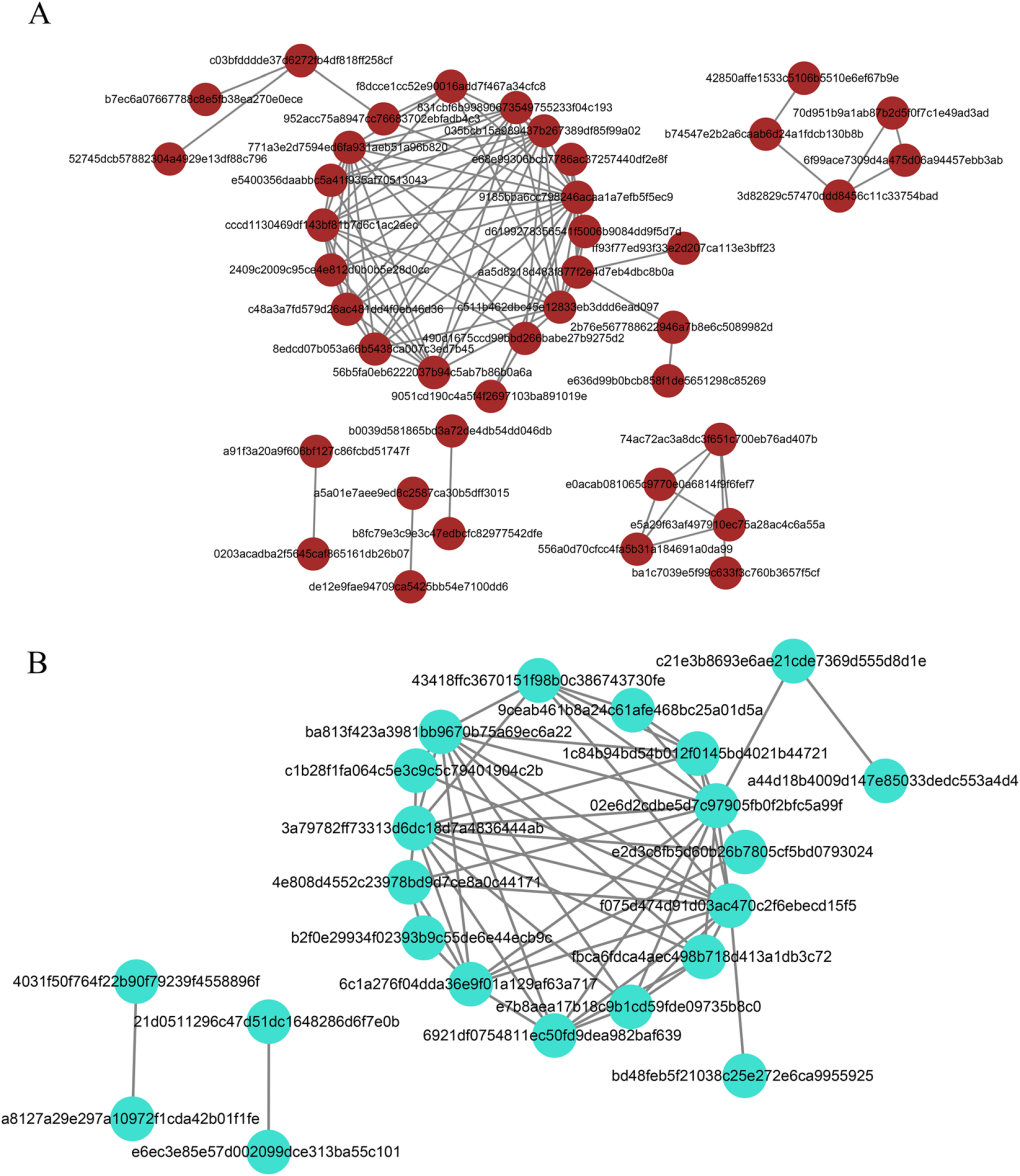
Microbial network diagram of key modules. (A) Brown module network. (B) Turquoise module network. Nodes represent microbial communities, whereas edges represent the interactions between microbial communities.

**Figure 6:**
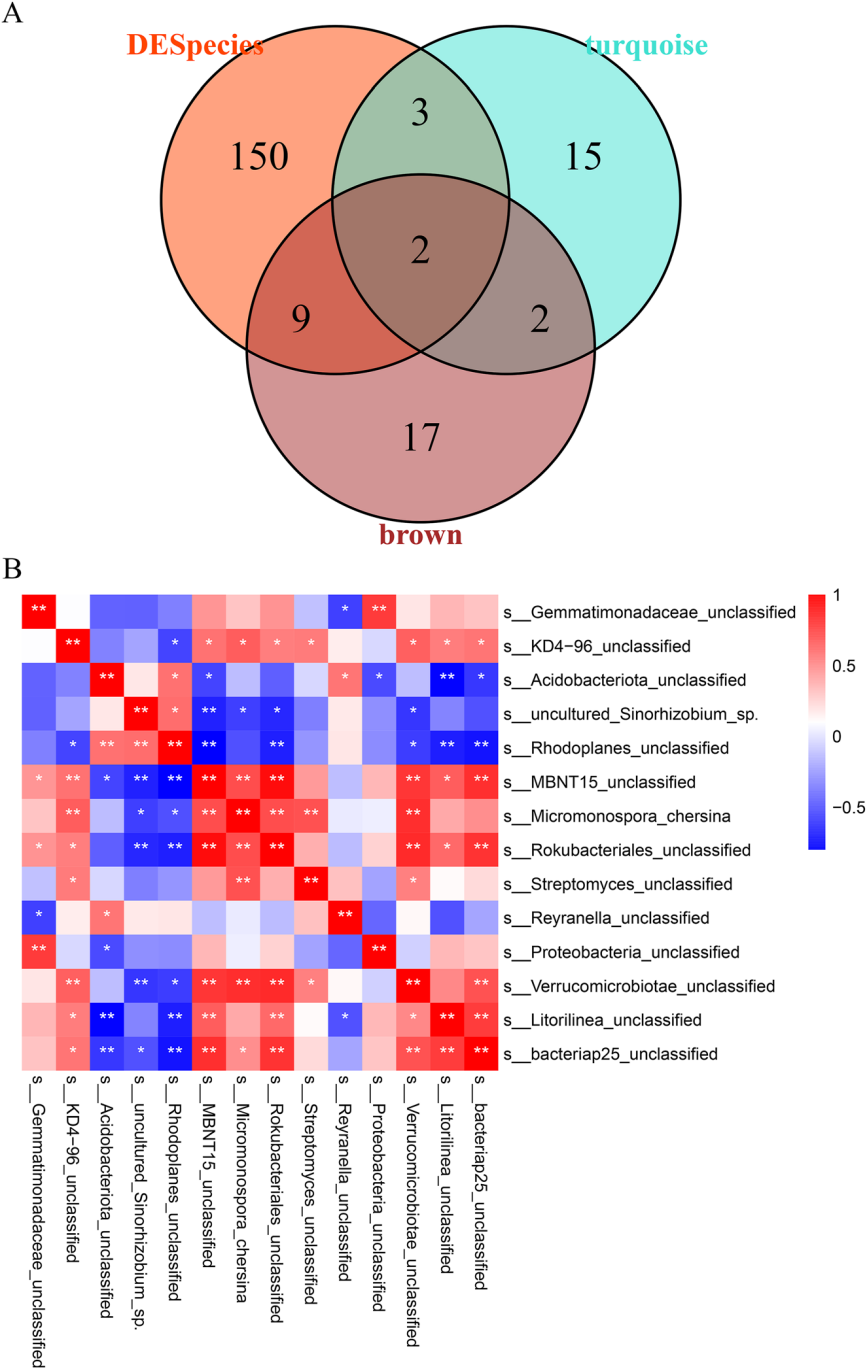
Screening and correlation analysis of hub microbiota. (A) Fourteen hub microbiota samples. The Venn diagram displays the core microbial community samples identified in different modules. Different colored regions represent different modules, and overlapping areas indicate microbial communities identified as key to multiple modules. (B) Correlation among hub microbiota. The heatmap displays the correlations between the 14 core microbial communities. Red represents a positive correlation, and blue represents a negative correlation. *: *P* < 0.05, **: *P* < 0.01.

To maximize the fulfillment of the scale-free network distribution premise, the “power” value was selected when the square value of the correlation coefficient reached 0.85 for the first time, which is “power” = 12 (Figure 4A). Nine modules were integrated (Figure 4B). In addition, the brown (*r* = 0.6 and *P* = 0.008) and turquoise modules (*r* = −0.55 and *P* = 0.02) were closely related to the regional groups (Figure 4C). The key module networks were constructed. The brown module network contained 40 nodes and 90 edges (Figure 5A), and the turquoise module network included 22 nodes and 55 edges (Figure 5B). After taking the intersection of the brown and turquoise modules with DEspecies, respectively, 14 hub microbiotas were identified at the species level (Figure 6A), including *Gemmatimonadaceae*, *KD4-96*, *MBNT15*, *Micromonospora chersina*, *Rokubacteriales*, *Streptomyces*, *Reyranella*, *Acidobacteriota*, *uncultured Sinorhizobium*, *Proteobacteria*, *Verrucomicrobiotae*, *Litorilinea*, *Rhodoplanes*, and *bacteriap25*. Finally, the correlation between the hub microbiota was evaluated (Figure 6B). Among which, *Gemmatimonadaceae* was positively correlated with *Proteobacteria*, whereas *Acidobacteriota* was negatively associated with *bacteriap25*.

## 4 Discussion

The microbial community in the roots of *P. chinensis (Bunge) Regel* should be studied because of its importance in the quality and value of medicinal materials and as it is affected by various factors, such as geography, climate, and cultivation practices. Previous studies have experimentally demonstrated that samples of *P. chinensis* from different regions of China can exhibit different qualities of medicinal properties [8], 25], 34], 35]. In this study, the microbial community composition of the roots was studied in *P. chinensis (Bunge) Regel* from three different regions, and the associations between the hub microbiota were explored. The microbial community in the roots of plants plays a pivotal role in determining the medicinal quality of its rhizomes, as evidenced by its involvement in secondary metabolite biosynthesis and abiotic stress tolerance [36], 37]. We found that, even in AH, DB, and HB, the microbial communities in the rhizosphere of several major regions of the Chinese *P. chinensis (Bunge) Regel* were different, resulting in different qualities of medicinal materials in each region.

The composition of the root microbiota of *P. chinensis (Bunge) Regel* exhibits distinct regional patterns, with variations in the abundance of specific phyla and genera across different geographical locations. This study shows that the phyla *Acidobacteriota, Firmicutes,* and *Gemmatimonadota* were enriched, and *Actinobacteriota, Chloroflexi*, and *Proteobacteria* were depleted in the root microbiota of *P. chinensis (Bunge) Regel* in DB; whereas *Actinobacteriota, Bacteroidota,* and *Proteobacteria* were enriched, and *Chloroflexi, Gemmatimonadota* were depleted in the root microbiota of *P. chinensis (Bunge) Regel* in AH, which is in line with the results of previous studies investigating the rhizosphere communities of numerous plant species [38], 39]. These studies emphasize the impact of environmental gradients on the rhizosphere microbiome of various plant species.

AH is a subtropical monsoon region with acidic soils and elevated heavy metal concentrations due to nearby industrial activity. The climate characteristics of high temperature and heavy rainfall, combined with acidic soil conditions, shape the rhizosphere microbial community dominated by *Actinobacteria*, *Bacteroidetes*, and *Proteobacteria*. Some strains of *Proteobacteria* have heavy metal tolerance and can reduce the bioavailability of heavy metals in soil by secreting organic acids, thereby alleviating heavy metal toxicity caused by industrial activities [40], 41]. The phylum *Actinobacteria* inhibits the growth of pathogenic bacteria and enhances plant resistance to diseases by producing antibiotics [42], 43]. *Bacteroidetes* participate in the decomposition of organic matter and provide usable nutrients for plants [44]. The synergistic effect of these bacterial strains enables the white haired Weng to maintain a good growth state in acidic and heavy metal-polluted environments. DB experiences a temperate continental climate and neutral soils. In the DB region, neutral soil and large temperature differences promote *Acidobacteriota*, *Firmicutes*, and *Gemmatimonadota* to become dominant bacterial species. *Acidobacteriota* play an important regulatory role in carbon cycling and can release nutrients by decomposing complex organic matter [45]. Some species in the *Firmicutes* phylum have nitrogen fixation ability and can increase soil nitrogen availability [46]. These functional characteristics are in line with the relatively stable nutrient cycling requirements in temperate regions, providing stable nutritional support for the *P. chinensis (Bunge) Regel*. HB represents a semi-arid zone with alkaline soils. In this region, *Proteobacteria* and *Gemmatimonadota* were dominant bacterial phyla in the rhizosphere microbiota of *P. chinensis (Bunge) Regel*. LEfSe analysis revealed that Rokubacteriales and its genus Methylomyrabilia were characteristic bacterial groups in the region, which may adapt to the alkaline soil environment of semi-arid areas through alkali-tolerant metabolic pathways. The negative correlation between core bacterial species may reflect the synergistic adaptation mechanism of microbial communities to the arid alkaline complex environment in the HB region. These findings suggested that soil pH, precipitation, and temperature can collectively explain changes in bacterial community structure, deepening our understanding of the regional differences in plant-microbe interaction mechanisms.

Fourteen hub microbiotas were identified at the species level: *Gemmatimonadaceae*, *KD4-96*, *MBNT15*, *M. chersina*, *Rokubacteriales*, *Streptomyces*, *Reyranella*, *Acidobacteriota*, *uncultured Sinorhizobium*, *Proteobacteria*, *Verrucomicrobiotae*, *Litorilinea*, *Rhodoplanes*, and *bacteriap25*. They may play a key role in the root microbiota of *P. chinensis (Bunge) Regel*. Gemmatimonadaceae has been identified as a core bacterium in the root microbiota of Tibetan hulless barley along altitudinal gradients [47], indicating its adaptability to diverse environmental condition. Peng et al. examined rhizosphere bacteria and soil functionality in response to different irrigation practices and found that *Gemmatimonadaceae*, which constitute the keystone taxa, were positively correlated with functional genes enriched in nutrient cycling [48]. This indicates their potential role in improving the nutritional availability of *P. chinensis (Bunge) Regel* in nutrient-limited environments. The unclassified Chloroflexi group *KD4-96*, which was abundant in the root microbiota of red clover [38], may also contribute to the microbial community structure of *P. chinensis (Bunge) Regel*, particularly in regions with specific soil types or farming systems [49]. The enrichment of *Rokubacteriales* by wheat roots under high levels of cadmium and arsenic highlights the potential of these bacteria to confer resistance to heavy metal stress, which could be relevant for *P. chinensis* growing in contaminated soils [50]. Santos-Medellín et al. showed that the most abundant endosphere taxon during drought and weeks after re-watering was *Streptomyces*, and a corresponding isolate promoted root growth [51]. *P. chinensis (Bunge) Regel* may maintain water balance during dry seasons by enriching Streptomyces [52], 53]. Reyranella, associated with smut resistance in sugarcane [54] and involved in denitrification processes [55], could contribute to disease resistance and nitrogen cycling in *P. chinensis (Bunge) Regel* rhizospheres. Wang et al. explored the efficacy of fosthiazate against root-knot disease in *Cucumis melo* var. *saccharinus* and its underlying role in the rhizosphere microbiome, and the results indicated *Acidobacteriota* as the predominant phylum [56]. Grunert et al. evaluated the microbial and fungal community structures in four different tomato cultivation systems, including both soil-based and soilless culture systems. The results showed that *Litorilinea* was the most crucial factor in distinguishing between natural soils supplemented with animal and plant byproducts [57], and has potential roles in soil organic matter decomposition and nutrient cycling, which may be related to the organic improvement of *P. chinensis (Bunge) Regel* in agricultural ecosystems. *Rhodoplanes*, predominant in cooled soils and contributing to lettuce growth maintenance at low temperatures [58], may also be beneficial for *P. chinensis (Bunge) Regel* in regions with cooler climates. Taken together, these findings suggested that the 14 hub microbiotas identified in *P. chinensis (Bunge) Regel* may be associated with a range of environmental and cultivation-related factors, including altitude, geography, climate, and soil management practices. The functional roles of these hub taxa in other plant systems provide valuable insights into their potential contributions to the microbiome dynamics and plant health of *P. chinensis (Bunge) Regel*.

This study is the first to investigate the microbial community composition of *P. chinensis (Bunge) Regel* roots in three different regions. However, this study had numerous limitations. First, the sample size was relatively limited, which may have masked rare microbial taxa (<0.1 % abundance) and weak ecotype-specific interactions. Small sample sizes limit the resolution of microbial co-occurrence networks, potentially obscuring the keystone taxa that orchestrate community assembly. Second, the sampling strategy of this study focused on three discrete regions with limited geographical coverage, ignoring key ecological areas for the growth of *P. chinensis (Bunge) Regel,* such as arid regions, coastal salt marshes, and alpine tundra regions. These omissions limit the ecological universality of our results, as they exclude extreme environmental filters that may drive rapid microbial evolution and co-diversification of host microorganisms. Finally, although this study elucidated the differences in regional microbial communities, it did not explore the molecular mechanisms underlying host-microbe interactions. Future research should expand the sample size and geographic coverage, and combine metagenomics of genome analysis with host transcriptomics to reveal microbial-mediated stress adaptation mechanisms, thereby linking ecological patterns with molecular driving factors.

## 5 Conclusions

In conclusion, information on the microbial community composition of the roots of *P. chinensis (Bunge) Regel* samples from three different regions is limited. This study provided basic information on the bacterial diversity and composition of *P. chinensis (Bunge) Regel* from three different regions. The 14 hub microbiota identified at the species level in this study may provide new insights into the cultivation of *P. chinensis (Bunge) Regel.*
